# Targeting HIV-1 reservoirs in T cell subsets

**DOI:** 10.3389/fimmu.2023.1087923

**Published:** 2023-01-20

**Authors:** Min Li, Marietta M. Budai, Min Chen, Jin Wang

**Affiliations:** ^1^ Immunobiology and Transplant Science Center, Houston Methodist Research Institute, Houston, TX, United States; ^2^ Department of Pathology and Immunology, Baylor College of Medicine, Houston, TX, United States; ^3^ Department of Surgery, Weill Cornell Medical College, Cornell University, New York, NY, United States

**Keywords:** HIV-1, cell death, anti-apoptotic molecules, autophagy, latency reversal agents, T cell subsets, humanized mice

## Abstract

The HIV-1 reservoirs harbor the latent proviruses that are integrated into the host genome. It is a challenging task to eradicate the proviruses in order to achieve an HIV cure. We have described a strategy for the clearance of HIV-1 infection through selective elimination of host cells harboring replication-competent HIV (SECH), by inhibition of autophagy and promotion of apoptosis during viral re-activation. HIV-1 can infect various CD4^+^ T cell subsets, but it is not known whether the SECH approach is equally effective in targeting HIV-1 reservoirs in these different subsets *in vivo*. In a humanized mouse model, we found that treatments of HIV-1 infection by suppressive antiretroviral therapy (ART) led to the establishment of latent HIV reservoirs in naïve, central memory and effector memory T cells. Moreover, SECH treatments could clear latent HIV-1 reservoirs in these different T cell subsets of humanized mice. Co-culture studies showed that T cell subsets latently infected by HIV-1, but not uninfected bystander cells, were susceptible to cell death induced by SECH treatments. Our study suggests that the SECH strategy is effective for specific targeting of latent HIV-1 reservoirs in different T cell subsets.

## Introduction

The human immunodeficiency virus (HIV) infects CD4^+^ T cells and myeloid cells (1–3). After entry into the host cells, the RNA genome of HIV-1 is reverse-transcribed into DNA and then integrated into host genome (4). The integrated HIV-1 provirus can remain dormant as latent proviruses that cannot be recognized by the immune system for clearance (5). When infected T cells undergo activation, cellular transcription factors are induced to bind to the 5′‐long terminal repeat (LTR) and promote the expression of HIV-1 genes (6). As a cytopathic virus, several proteins of HIV-1, such as Tat, Nef, Vif and Vpr, have been shown to induce T cell death (7–14). Without intervention by antiretroviral therapy (ART), severe depletion for CD4^+^ T cells by HIV-1 can lead to the clinical onset of the acquired immunodeficiency syndrome. While ART can efficiently reduce viral load to undetectable level and significantly extend the lifespan of people living with HIV, stable HIV-1 proviral reservoirs persist during ART treatments (15–17).

Previous studies suggest that T cell reservoirs of HIV type 1 (HIV-1) display the phenotypes characteristic of long-lived memory T cells (18–21). The cellular properties of longevity and quiescence of memory T cells could therefore be exploited to eliminate HIV-1 reservoirs. We and others have shown that the long-lived memory T cells and memory B cells protect their longevity by employing pro-survival autophagy as well as cellular anti-apoptotic machineries (22–26). Based on our experience in studying the long-term survival of memory T and memory B cells (22), we investigated whether targeting cellular survival mechanisms may promote the clearance of HIV reservoirs. While memory T cells display active autophagy and express high levels of anti-apoptotic molecules, we found that a latency reversal agent (LRA), ingenol-3,20-dibenzoate (IDB), further promoted autophagy and increased the expression of anti-apoptotic molecules (27). Mechanistically, transcription factors involved in HIV-1 gene expression from the 5′‐LTR, such as NF‐κB, NFAT and HIF‐1α, and AP‐1 (28–33), are also important for the expression of cellular anti-apoptotic and autophagy genes (34). In order to facilitate the killing of reservoirs during viral re-activation, it would be important to suppress pro-survival autophagy and anti-apoptotic molecules that are upregulated during viral re-activation. We therefore developed an approach to delete HIV-1 reservoirs by selective elimination of host cells harboring replication-competent HIV (SECH), through the inhibition of pro-survival autophagy and anti-apoptotic molecules induced by latency reversal (27). The SECH strategy allows efficient killing of infected cells by induced HIV-1 proteins during viral re-activation, through suppression of cellular pro-survival mechanisms induced by latency reversal (27).

While latent HIV-1 was first detected in resting memory T cells (18–21), HIV-1 reservoirs are found in CD4^+^ T cells at different differentiation stages (35–38). CD4^+^ T effector cells with different profiles of cytokine production or functions are also susceptible to HIV-infections. The types of CD4^+^ T cell subsets and tissue origins of infections have only limited influences on HIV-1 integration (39). Effector memory T cells expressing more CCR5 show higher percentages of infection by HIV-1 type C, but all CD4 T cell subsets could be infected (40). CD4^+^ T effector cells that secrete different profiles of cytokines, including Th0, Th1, Th2 cells, Th17 cells, follicular T helper cells (Tfh) and follicular T regulatory cells (Tfr) are all susceptible to HIV-1 infection (41–43). Additionally, CD4^+^ T cells with specificities against different HIV-1 proteins are similarly infected by the virus (44). Using more robust methods for the detection of intact HIV-1 DNA, latent HIV-1 can be detected in different T cell subsets with resting phenotypes, including naïve and memory T cells (45). Moreover, homeostatic proliferation of T cells infected by HIV-1 may lead to the clonal expansion of certain HIV-1 reservoirs in patients (20, 46–48). It is therefore necessary to target HIV-1 in all T cell subsets at different differentiation stages in order to achieve viral clearance.

Accumulating evidence suggests that HIV-1 can infect T cells at different differentiation stages, and establish latent reservoirs in various T cell subsets. However, *in vivo* studies for the susceptibility of HIV reservoirs in various T cell subsets to HIV clearance are still lacking. Here we established HIV-1 latent infection in T cell subsets in a humanized mouse model. Our study suggests that it is possible to target HIV reservoirs in CD4^+^ T cells at various developmental stages to achieve the elimination of latent HIV-1 reservoirs in humanized mice.

## Materials and methods

### HIV-1 cure studies in humanized mice

Humanized mice used in this study were generated by reconstituting NSG-SGM3 mice (The Jackson Laboratory) with CD34^+^ human stem cells as described previously (27). Three months after reconstitution with human stem cells, flow cytometry analyses were performed to examine human immune cells in mouse peripheral blood. The mice were then injected with HIV-1 (AD8 strain, 1000 pfu/mouse, i.p.). Ten days post-infection, peripheral blood was collected and RNA was extracted using the MagMAX-96 Blood RNA Isolation Kit (Thermo Fisher Scientific), followed by real-time PCR for Pol-1 and LTR-gag to confirm HIV-1 infections (27). The mice were then treated by ART with raltegravir (10 mg/kg b.w., Adooq Bioscience), BMS-663068 (10 mg/kg b.w., Adooq Bioscience) and lamivudine (15 mg/kg b.w., Macleods Pharma.) orally to suppress viral replication. After treatment with suppressive ART for 40 days, the mice were changed to treatments by SECH regimen, including raltegravir (10 mg/kg b.w.), BMS-663068 (10 mg/kg), IDB (1.2 mg/kg, ENZO Life Sciences), ABT-263 (25 mg/kg) and SAR405 (25 mg/kg) with or without JQ1 (12 mg/kg) (from MedChemExpress) dissolved in the solvent containing ethanol (10%), polyethylene glycol 400 (30%; Sigma) and Phosal 50 PG (60%; Fisher Scientific), once every two days. Control mice are treated with raltegravir and BMS-663068 for ART only. This study was conducted according to federal and institutional guidelines, and was approved by the Institutional Animal Care and Use Committee of the Houston Methodist Research Institute. After SECH treatments for 35 cycles, mice were left untreated for 2 months. Peripheral blood before and after withdrawal of the treatments was collected to quantitate HIV-1 mRNA. Spleen cells were harvested, and red blood cells were lysed with ammonium chloride lysing buffer (0.15 M NH4Cl, 10 mM KHCO3, 0.1 mM EDTA). The cells were then stained with antibodies for different CD4^+^ T cell subsets. CD45RA^+^CD45RO^-^CCR7^+^ naïve T cells, CD45RA^–^CD45RO^+^CCR7^+^ CMT and CD45RA^–^CD45RO^+^CCR7^–^ EMT were sorted using a FACSAria cell sorter (BD Biosciences). Bone marrow cells were also isolated from femur and tibia as described (49). Viral rebound in those cell subsets were also analyzed by quantification of HIV-1 mRNA.

### Flow cytometry

Antibodies from Biolegend that were used in this study include: APC-anti-human CD45 (1:100, 304012, clone HI30), Pacific Blue-anti-mouse CD45 (1:100, 103126, clone 30-F11), PE-anti-human CD4 (1:100, 317414, clone OKT4), Pacific Blue-anti-human CD19 (1:100, 302232, clone HIB19), Pacific Blue-anti-human CD3 (1:100, 300329, clone HIT3a), APC/Fire-750-anti-human CD8 (1:100, 34474, clone SK1), APC/Fire-750-anti-human CD163 (1:100, 333633, clone GHI/61), PE/Cy7-anti-HLA-HLA-DR, DP, DQ (1:100, 361708, clone Tü39), PerCP/Cy5.5-anti-human CD3 (1:100, 300328, clone HIT3a), PE-anti-human CD123 (1:100, 306005, clone 6H6), PerCP/Cy5.5 anti-human CD123 (1:100, 306016, clone 6H6), FITC-anti-mouse CD45 (1:100, 103108, clone 30-F11), PE/Cy7 anti-human CD197 (CCR7) (1:100, 353226, clone G043H7), PE-anti-human CD45RO (1:100, 304244, clone UCHL1), FITC-anti-human CD45RA (1:100, 304148, clone HI100), Alexa Fluor 488-anti-CD68 (1:30, 333812, clone Y1/82A), PerCP/Cy5.5-anti-human CD19 (1:100, 302230, clone HIB19), PE-anti-human CD27 (1:100, 356406, clone M-T271) and Pacific Blue-anti-human IgD (1:100, 348224, clone IA6-2). The cells were stained with antibodies for cell surface markers and flow cytometry was performed using a BD LSR II cytometer (BD Biosciences). To detect HIV-1 p24, the cells were first stained with antibodies specific for T cell subset makers. The cells were then fixed and permeabilized with the Cytofix/Cytoperm buffer (BD Bioscience). Intracellular staining for HIV-1 p24 was then performed using PE-conjugated anti-p24 (1:30, 6604667, clone KC57, from Beckman Coulter). Flow cytometry was carried out with a BD LSR II flow cytometer (BD Biosciences). The data were analyzed by the FlowJo software (BD Biosciences).

### Quantification of HIV-1 mRNA

Peripheral blood was collected (50 to 100 μl/mouse), and mRNA from whole blood samples was extracted using the MagMAX-96 Blood RNA Isolation Kit (Thermo Fisher Scientific). Alternatively, RNA was isolated from purified T cells with MagMAX-96 for Microarrays Total RNA Isolation Kit (ThermoFisher Scientific). mRNA was then converted to cDNA with the SuperScript VILO cDNA Synthesis Kit (ThermoFisher Scientific), followed by real-time quantitative RT-PCR (qRT-PCR) for LTR-GAG or HIV-1 pol-1 according to our previous procedure (27).

### TZA assay

TZM-bl cells (NIH AIDS Reagent Program) were seeded in 96 well plates (60,000 cells/well) and cultured for 24 hours. Virus outgrowth by the TZA assay was performed as described (30). Spleen cells (5x10^6^/sample) from Hu-HSC mice were stimulated with anti-CD3/CD28-Dynabeads (ThermoFisher Scientific) for 48 h, followed by co-cultured with TZM-bl cells for another 48 h in the presence of 5 μg/ml PHA, 0.1 μg/ml LPS and 100 nM CpG. Beta-galactosidase activity was determined using the Beta-Glo Assay System (Promega).

### T cell isolation and infection with HIV-1

CD4^+^ T cells were purified from the peripheral blood mononuclear cells (PBMCs) of healthy donors at the Gulf Coast Blood Center using anti-CD4 MACS beads (Miltenyi Biotec). CD3^+^CD4^+^CD45RA^+^CD45RO^-^CCR7^+^ naïve T cells, CD3^+^CD4^+^CD45RA^–^CD45RO^+^CCR7^+^ central memory T cells (CMT) and CD3^+^CD4^+^CD45RA^–^CD45RO^+^CCR7^–^ effector memory T cells (EMT) were sorted on a BD FACSAria flow cytometer (BD Bioscience). The cells were infected with HIV-1 CXCR4-tropic NL4-3 (1 MOI) as described (27), followed by culture with CCL19 (30 nM, Biolegend) and IL-2 (0.3 ng/ml, Biolegend) for 4 days to establish latent HIV-1 infection (27).

### Induction of cell death

Sorted T cell subsets with latent HIV-1 infection as above were then stimulated with 0.1 μM ingenol-3,20-dibenzonate (IDB, ENZO Life Sciences). ABT-263 (0.2 μM, Adooq Bioscience) and SAR405 (2 μM, MedChemExpress) were added as indicated. After culture for 48 h, the cells were incubated with FITC-DEVD-FMK, followed with APC-Annexin V (Biolegend). The cells were then used for intracellular staining of p24 as above and analyzed by flow cytometry. Percentages of cell death were calculated by the loss of viable cells negative for annexin V and DEVD staining: (untreated – treated)/untreated x 100%.

To determine the specificity in the killing of HIV-1 infected cells, uninfected T cell subsets were labeled with CellTrace Violet dye (ThermoFisher Scientific) and mixed with corresponding HIV-1-infected T cell subsets at the ratio of 1:1. The cells were cultured with 0.2 μM BMS-626529, 0.1 μM IDB, 0.2 μM ABT-263 and 2 μM SAR405 as indicated for 48 h. The cells were incubated with DEVD-FMK and Annexin V as above and analyzed by flow cytometry.

### Statistical analyses

Statistical analyses were performed using GraphPad Prism 8 software. Data were shown as mean ± SD and statistics analysis was performed by two-tailed Student’s t-test or one way ANOVA. Significant statistical differences (*P*< 0.05 or *P*< 0.01) are shown in the Figures.

## Results

### Establishment of HIV-1 latent infection in T cell subsets of humanized mice

**Figure 1:**
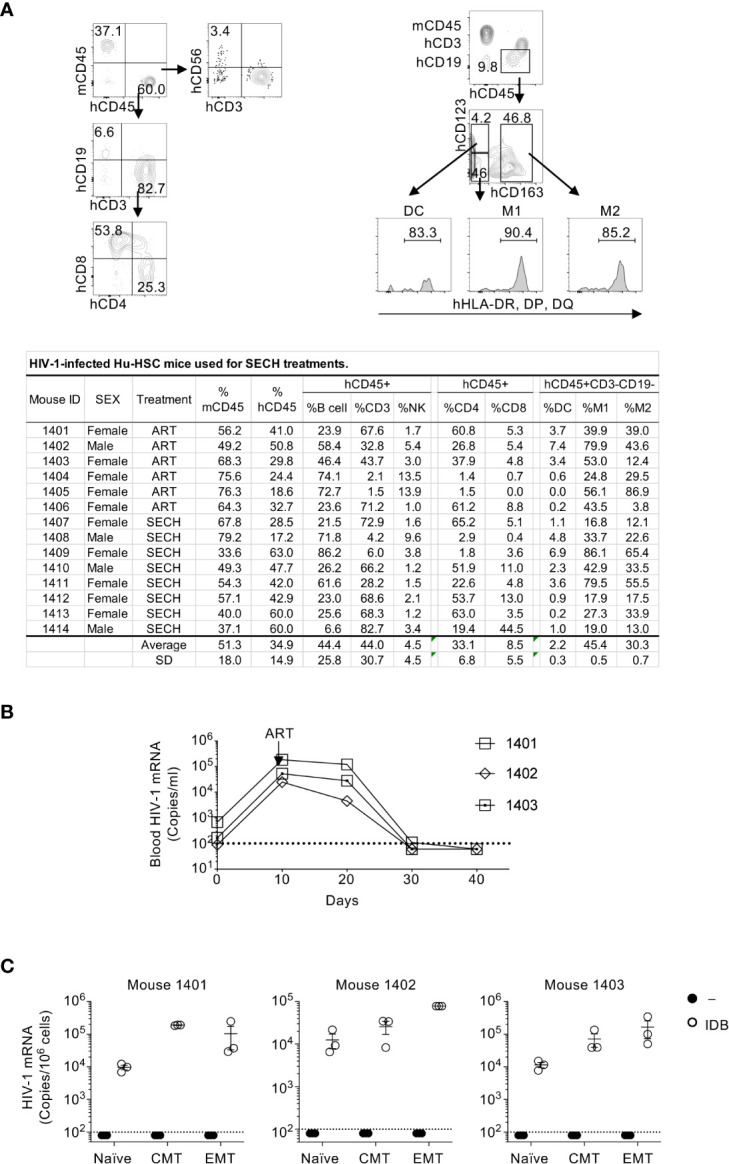
HIV-1 latent infection in human T cell subsets of Hu-HSC mice. **(A)** Hu-HSC mice were produced by reconstituting NSG-SGM3 mice with human CD34^+^ hemopoietic progenitor cells. Three months after implantation of CD34 cells, human immune cells positive for human CD45 (hCD45^+^) and negative for mouse CD45 (mCD45^–^) in the peripheral blood of Hu-HSC mice was quantified by flow cytometry. The representative plot of flow cytometry of Hu-HSC was shown in upper penal, and the quantification of immune cell populations from each individual mouse was presented in the lower panel. **(B)** Hu-HSC mice (n=3) were infected with HIV-1 (AD8 strain, 1000 pfu/mouse), followed by ART treatment on day 10 post infection. Peripheral blood was collected, and HIV-1 mRNA in the whole blood of Hu-HSC mice was determined by RT-PCR. **(C)** Human T cell subsets were sorted from mice in **(B)** after ART treatments, and re-stimulated with IDB for 24 h, following by measurement of HIV-1 mRNA by RT-PCR. The dash line indicates detection limit.

Immunodeficient NSG-SGM3 mice reconstituted with human CD34^+^ hemopoietic progenitor cells (Hu-HSC) were used for our HIV infection and cure studies. These Hu-HSC mice showed efficient development of human CD45^+^ (hCD45^+^) CD4^+^ and CD8^+^ T cells, CD19^+^ B cells, CD3^–^CD56^+^ NK cells, CD123^+^CD163^–^MHC-II^+^ dendritic cells, as well as CD123^–^CD163^+^MHC-II^+^ and CD123^–^CD163^–^MHC-II^+^ macrophages (**Figure 1A**). At day 10 after HIV-1 infection, treatment with the ART regimens, including raltegravir, BMS-663068 and lamivudine (50–52), were used to inhibit HIV-1 replication and establish latent infection (27). ART efficiently suppressed HIV-1 virus in the peripheral blood after 30-40 days of treatments (**Figure 1B**).

We could detect HIV-1 reservoirs in the spleen, bone marrow, liver, lung and kidney of HIV-1-infected humanized mice (27), suggesting that all of these sites contain HIV-1-infected cells. However, NSG mice reconstituted with human hematopoietic stem cells display severe hypoplasia in different lymph nodes (53) and have very few human intestinal T cells (54). To obtain sufficient number of different subsets of T cells, we therefore isolated T cell subsets from the spleen of HIV-1-infected humanized mice in this study. We then sorted human naïve T cells, central memory T cells (CMT) and effector memory T cells (EMT) from these mice. As expected, HIV-1 mRNA was undetectable in unstimulated naïve T cells, CMT and EMT purified from ART-treated Hu-HSC mice (**Figure 1C**). However, viral reactivation with IDB induced the expression of HIV-1 mRNA in these T cell subsets (**Figure 1C**). These results demonstrate that HIV-1 latent infection can be established in different T cell subsets, and that the latent HIV-1 can be efficiently reactivated by IDB.

### Clearance of HIV-1 reservoirs in T cell subsets *in vivo* by SECH treatments in humanized mice

**Figure 2:**
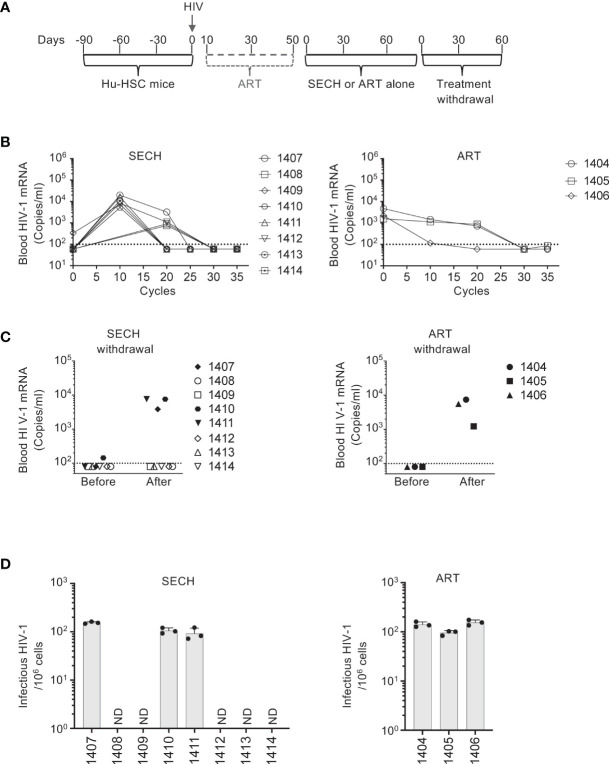
Clearance of HIV-1 infection in Hu-HSC mice. **(A)** A schematic diagram for SECH treatment of HIV-1-infected Hu-HSC mice. Three months after reconstitution with human CD34^+^ hemopoietic stem cells, Hu-HSC mice were infected with HIV-1 (AD8 strain, 1000 pfu/mouse), followed by suppressive ART treatments. After 40 days of suppressive ART, the mice were then treated by SECH or ART (35 cycles, 70 days), followed by withdrawal of treatments for 2 months. **(B)** HIV-1-infected mice were treated by SECH or ART alone (35 cycles). HIV-1 mRNA in the peripheral blood was determined as in **Figure 1B**. **(C)** ART and SECH treatments in **(B)** were withdrawn for 2 months. HIV-1 mRNA in the peripheral blood before and after withdrawal of treatment was determined. **(D)** Infectious HIV-1 in splenocytes from Hu-HSC miceafter withdrawal of treatments **(C)** was measured by TZA assays. ND, not detectable.

We then determined the susceptibility of latent HIV-1 reservoirs in T cell subsets to SECH treatments *in vivo*. Different from our previous study in which SECH treatments were started at day 10 after HIV infection without the suppressive ART (27), here we treated Hu-HSC mice with suppressive ART, including BMS-663068, raltegravir, and lamivudine (50–52) to inhibit active viral replication (**Figure 2A**). This suppressive ART treatment potentially promotes the establishment of latent HIV reservoirs in T cell subset as shown in **Figure 1**. After 40 days of ART treatment, HIV-1 mRNA levels became low or undetectable in the peripheral blood of these Hu-HSC mice. The mice were then treated with SECH regimens (27), including: 1) IDB and JQ1 as LRAs; 2) ABT-263 (55), a Bcl-xL antagonist to inhibit apoptosis; and 3) SAR405, an autophagy inhibitor for class III PI3 kinase VPS34 in the Beclin 1 signaling complex (56). Only raltegravir and BMS-663068 were included as ART components to block new infections, without preventing the production of novel viral proteins and virions (27). The control group received ART alone. These treatments were given once every two days as one cycle (**Figure 2A**). After 35 cycles of SECH treatment, HIV-1 mRNA was undetectable in the mice treated by SECH (**Figure 2B**). We then assessed viral rebound by withdrawal of treatments for two months. After withdrawal of SECH, viral rebound was found in the peripheral blood of some mice (3 out of 8 mice), while HIV-1 mRNA remained undetectable in the other mice (**Figure 2C**). While ART can efficiently reduce viral load to undetectable level and significantly extend the lifespan of people living with HIV, a stable HIV-1 proviral reservoir persists during cART, and continuous cART is necessary to prevent new virus production from the HIV-1 reservoirs (15–17). Consistent with the failure for ART to clear HIV in our previous studies (27), all 17 mice treated in our previous study (27) and the mice treated in this study by ART alone **(** **Figure 2C**) showed HIV-1 rebound after treatment interruption. We next isolated the spleen cells from mice with or without HIV-1 rebound and measured infectious HIV-1 by TZA virus outgrowth assay. Consistently, we found that spleen cells purified from SECH-treated mice with HIV-1 rebound produced infectious viruses, whereas spleen cells from mice without HIV-1 rebound produced no infectious viruses (**Figure 2D**). Our previous studies show that HIV-1 is successfully cleared in the spleen, bone marrow, lung, liver and kidney (27). This suggests that SECH is effective for targeting all HIV-1 reservoirs in different organs and tissues, potentially in both T cells and myeloid cells.

**Figure 3:**
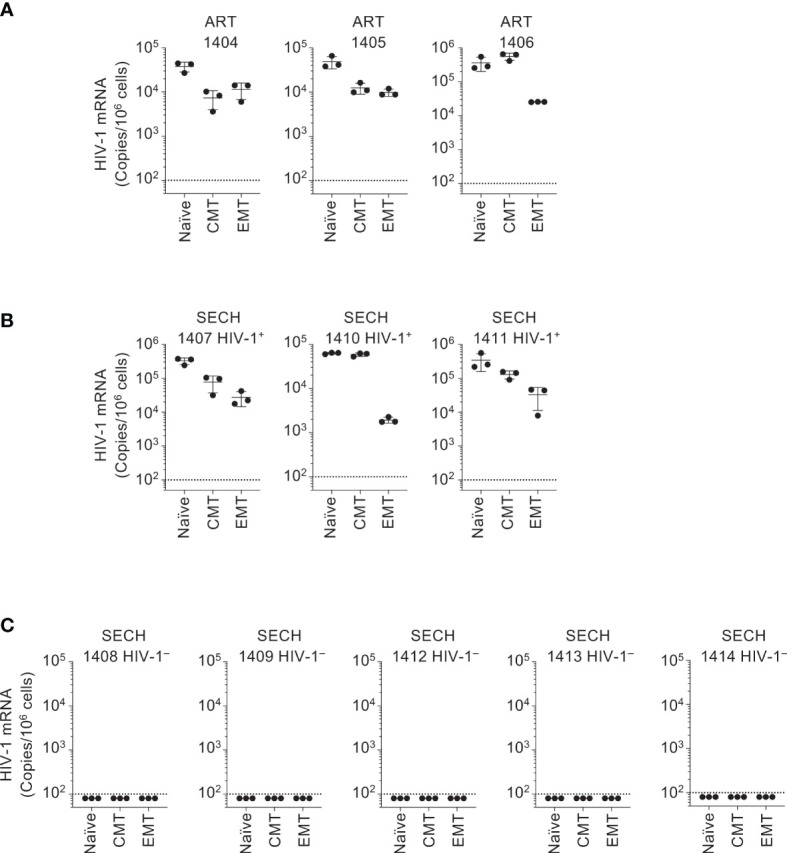
Clearance of HIV-1 reservoirs in T cell subsets of Hu-HSC mice by SECH. Human CCR7^+^CCD45RO^–^ naïve CD4^+^ T cells, CCR7^+^CCD45RO^+^ CMT and CCR7^–^CCD45RO^+^ EMT were sorted from Hu-HSC mice infected with HIV-1 that had been treated by ART **(A)**, and from the mice with **(B)** or without **(C)** HIV-1 rebound after withdrawal of SECH treatment. HIV-1 mRNA levels in the cells were determined by qRT-PCR.

In our previous study of SECH treatments of HIV-1-infected humanized mice, viral clearance was demonstrated in total blood, spleen and bone marrow cells, as well as in the lung, liver and kidney (27). However, HIV-1 clearance in T cells or subsets of T cells sorted from these mice was not studied. To determine whether HIV-1 was cleared in different T cell subsets by SECH, we sorted T cell subsets from the spleens of the mice with or without HIV-1 rebound. In ART-treated mice, HIV-1 mRNA was detected in naïve T cells, CMT, and EMT after treatment withdrawal (**Figure 3A**), suggesting that ART alone cannot clear HIV-1 infections in different T cell subsets. HIV-1 mRNA was also detected in naïve T cells, CMT, and EMT from SECH-treated mice that showed HIV-1 rebound (**Figure 3B**). This indicates that the failure to respond to SECH treatments is not unique to a specific T cell subset. Importantly, HIV-1 mRNA was not detectable in T cell subsets sorted from SECH-treated HIV-1 negative mice (**Figure 3C**). These data indicate that HIV-1 infections in different T cell subsets can be successfully eradicated by SECH in humanized mice.

In our previous of treatments of HIV-1-infected humanized mice, SECH treatments initiated at day 10 post-infection led to a relative decrease in naïve T cells and an increase in effector memory T cells (27). In the current study, suppressive ART treatments were started at day 10 post-infection for 40 days, and SECH was started when HIV-1 became undetectable in the peripheral blood to better mimic people living with HIV under long-term ART treatments (**Figures 1B**, **2A**). Interestingly, similar results of the decrease in naïve T cells and the increase in effector memory T cells were observed in SECH-treated mice after initial suppressive ART treatments (**Supplementary Figure 1**). This indicates the SECH treatments induce a certain degree of T cell activation in Hu-HSC mice with or without prior exposure to suppressive ART.

### Comparable sensitivity to SECH treatment among T cell subsets

**Figure 4:**
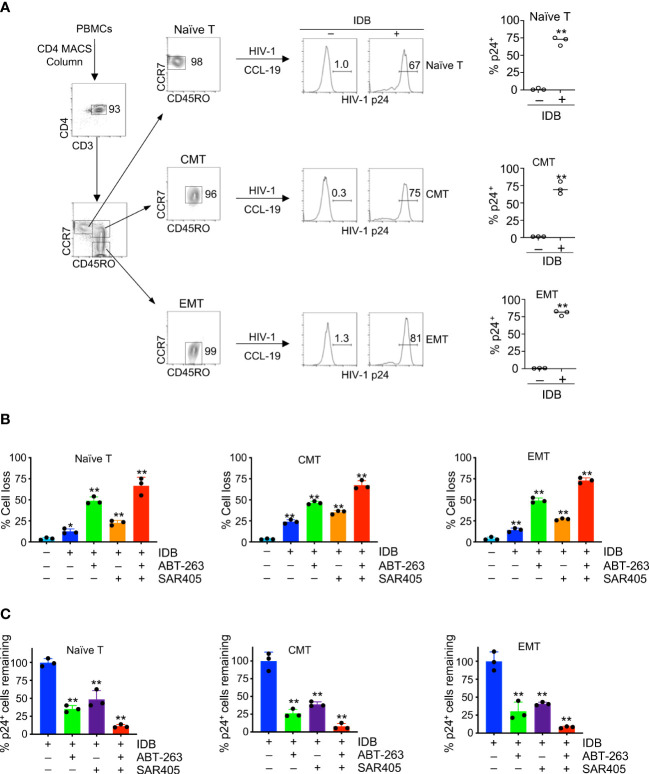
Induction of cell death in HIV-1-infected naïve, central memory and effector memory T cells. **(A)** CD4^+^ T cells were purified from human PBMCs using anti-CD4-MACS beads (Miltenyi Biotec), and naïve, central memory (CMT) and effector memory T cells (EMT) were sorted by flow cytometry. Sorted T cell subsets were infected with HIV-1 (NL4-3, 1 MOI). Latent infection was then established by culturing with CCL-19 for 4 days, followed by stimulation with IDB for 24 h. Intracellular staining of HIV-1 p24 was determined by flow cytometry. **(B, C)** The cells with or with latent HIV-1 infection as in **(A)** were treated for 48 h with IDB (0.1 μM), ABT-263 (0.2 μM) and SAR405 (2 μM) as indicated. The cells were then stained with DEVD-FITC and APC-Annexin V, followed by intracellular staining with PE-anti-HIV p24. Total cell death for cells **(B)** and the remaining viable HIV-1 p24^+^ cells negative for staining by APC-annexin V and DEVD-FITC **(C)** were calculated. Data represent biological replicates and are presented as mean ± SD. *P* values was determined by unpaired, two-tailed *t* test in **(A)**, or by one-way ANOVA with unpaired, two-tailed *t*-test in **(B)** and **(C)** (Control *vs.* different treatments). ** *P*<0.01.

We further verified the responsiveness of HIV reservoirs in different T cell subsets to SECH treatments. We have previously demonstrated that latent HIV-1 infection could be established in CMTs after *in vitro* culture with CCL-19 and reactivated by stimulation with phytohaemagglutinin (PHA) or IDB (27). However, this was not directly demonstrated in other T cell subsets. We first determined whether latent HIV-1 reservoirs could be efficiently established in CD4^+^ T cells at different stages of T cell development *in vitro*. We sorted naïve T cells, CMT and EMT (**Figure 4A**) from human peripheral blood monocytes (PBMC). After infection with HIV-1 at 1 multiplicity of infection (MOI), the cells were cultured in the absence of cytokines, but with CCL19 to sustain cell survival and establish latent HIV infections (57). While HIV-1 p24 was undetectable in these naïve T cells, CMT and EMT without stimulation, p24 was found in these T cell subsets after stimulation with IDB (**Figures 4A**). These data suggest that latent HIV-1 infection could be established in different T cell subsets, and the responses to LRAs for viral reactivation are similar in these subsets.

We determined whether different T cell subsets latently infected by HIV-1 could undergo similar levels of cell death after latency reversal. Stimulation of HIV-1-infected naïve T cells with IDB induced cell death after two days of culture (**Figure 4B**, **Supplementary Figure 2**). Similar results were observed for CMT and EMT with latent HIV-1 infection (**Figure 4B**, **Supplementary Figure 2**). Addition of ABT-263 or SAR405 increased cell death in HIV-1-infected T cell subsets stimulated with IDB (**Figure 4B**, **Supplementary Figure 2**), and reduced HIV-p24-expressing cells (**Figure 4C**). Furthermore, using ABT-263 together with SAR405 induced more killing of naïve T cells, CMT and EMT infected by HIV-1 (**Figure 4B**, **Supplementary Figure 2**), and caused most reduction of HIV-p24-expressing cells (**Figure 4C**). Consistent with our previous observations (27), these data indicate that the SECH treatment can sensitize different T cell subsets to cell death. Targeting autophagy and anti-apoptotic molecules further increases the killing in HIV-1-infected T cell subsets with a comparable efficacy.

### The specificity for SECH in the killing of HIV-1-infected naïve T Cells, CMT and EMT

**Figure 5:**
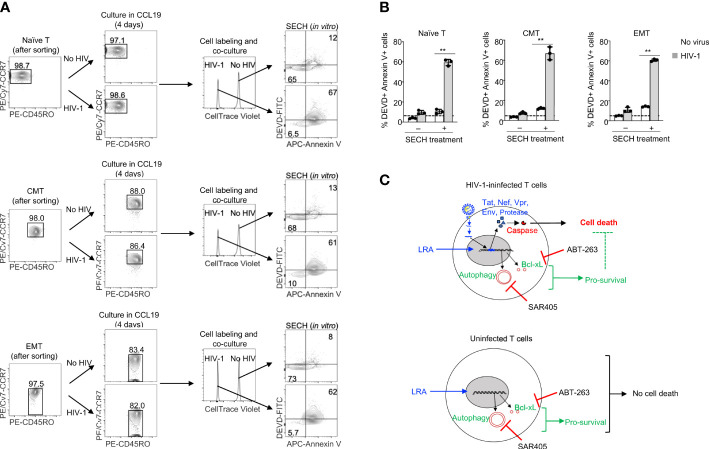
Specificity in the killing of HIV-1-infected naïve T cells, CMT and EMT, but not uninfected bystander cells. **(A, B)** HIV-1 latent infection was established in naïve T cells, CMT and EMT as in **Figure 4**, and mixed with CellTrace Violet-labeled uninfected counterparts. Cells were cultured with SECH components (IDB, ABT-263 and SAR405) in the presence of BMS-626529 as indicated for 48 h. The cells were then stained with DEVD-FITC and APC-Annexin V, and analyzed by flow cytometry **(A)**. Percentages of DEVD-FITC^+^ Annexin V^+^ cells were quantified **(B)**. Data represent biologically replicates and are presented as mean ± SD. Comparison with uninfected cells: **P<0.01. **(C)** Viral reactivation with LRAs can induce the expression of HIV-1 components that trigger apoptosis. However, LRAs simultaneously upregulate pro-survival autophagy and anti-apoptotic molecules that counteract viral product-induced cell death, resulting in incomplete killing of HIV-1-infected cells. Inhibiting pro-survival autophagy and anti-apoptotic molecules allows viral products to induce high levels of cell death after treatments with LRAs. In uninfected bystanders, upregulation of pro-survival autophagy and anti-apoptotic molecules by IDB would confer protection against cell death.

We next determined the specificity of SECH in the killing of different T cell subsets with HIV-1 infection in co-cultures with uninfected cells. Naïve T cells, CMT and EMT were infected with HIV-1 and cultured in the presence of CCL19 to establish HIV-1 latency. While naïve T cells with or without HIV-1 infection retained their CD45RO^–^CCR7^+^ surface phenotypes, CMT (CD45RO^+^CCR7^+^) and EMT (CD45RO^+^CCR7^–^) showed some decreases in CCR7 expression after culture (**Figure 5A**). To determine the specificity in the killing of HIV-1-infected cells, we labeled uninfected naïve T cells, CMT and EMT with Celltrace Violet, and co-cultured these cells with the corresponding subsets of T cells latently infected by HIV-1. The cells were then treated with SECH regimen, including IDB, ABT-263 and SAR4505. An HIV-1 attachment inhibitor, BMS-626529 (58), was also included to prevent new rounds of viral infections. The combination of latency reversal with inhibition of pro-survival autophagy and anti-apoptotic molecules efficiently induced cell death in HIV-1-infected naïve T cells, as shown by Annexin V staining and DEVD cleavage (**Figures 5A, B** **)**. In contrast, uninfected naïve T cells in the co-culture were relatively resistant to cell death (**Figures 5A, B** **)**. Similar results for specific killing of HIV-1-infected cells, but not uninfected controls in the co-culture, were observed for CMT and EMT (**Figures 5A, B** **)**. These co-culture studies suggest that naïve T cells, CMT and EMT infected by HIV-1 are susceptible to killing by SECH treatment, while uninfected T cells are relatively resistant to the SECH-mediated cell death (**Figure 5C**).

## Discussion

This study demonstrates that HIV-1 latent infection could be established in naïve T cells, CMT and EMT *in vivo* in a humanized mouse model. Furthermore, treatments by SECH can clear HIV-1 infections in all of these T cell subsets. Co-culture studies showed that the SECH treatments killed HIV-1-infected naïve T cells, CMT and EMT, but not uninfected bystander cells, suggesting that SECH is specific in targeting different subsets of T cells infected by HIV-1, but not their uninfected counterparts. This study suggests that SECH is effective in specifically targeting the HIV-1 reservoirs in different T cell subsets both *in vitro* and *in vivo*. SECH treatments in our previous study was started at day 10 post-infection with prior suppressive ART for treating acute HIV infections (27). In this study, suppressive ART before SECH treatment potentially promotes the establishment of latent HIV reservoirs in T cell subset (**Figure 1C**), thus better mimicking treating chronic infections in people living with HIV who have undergo long-term ART treatments. Taken together, the current work and our previous study (27) suggest that the SECH regimen can effectively clear HIV infected cells in humanized mice at both acute (without ART) and chronic stages of infection (on suppressive ART).

Re-activation of integrated DNA requires cellular transcription factors to drive HIV-1 gene expression from the 5’-LTR (28). These cellular factors that bind to HIV-1 LTR, such as AP-1, NF-κB and NFAT, can also induce the expression of cellular genes that are important for promoting cell survival and cellular metabolism, including anti-apoptotic Bcl-xL and Mcl-1, as well as autophagy-related genes (29–33). Cell death triggered by the expressed HIV-1 proteins can thus be suppressed by these induced cellular survival mechanisms (34). Therefore, the anti-apoptotic and autophagy genes up-regulated by LRAs during viral reactivation should be inhibited in order to unleash cell death signaling triggered by the expressed HIV-1 genes (**Figure 5C**). In uninfected cells, by contrast, anti-apoptotic and autophagy genes induced by LRAs likely confer the protection of uninfected bystander cells from SECH treatments (**Figure 5C**). SECH therefore can maximize cell death induced by HIV-1 genes in infected cells, through counteracting the pro-survival mechanisms induced by latency reversal, including anti-apoptotic molecules and autophagy. Consistent with our previous observations for the specificity of SECH in the killing HIV-1-infected cells (27, 34), the current study suggests that SECH is potentially safe to kill HIV-1-infected T cell subsets specifically, but not uninfected cells.

We and other have demonstrated that autophagy is critical for the long-term persistence of memory T and memory B cells (22–26). This raised the questions whether the memory cell populations are particularly vulnerable to the SECH treatment, as it involves the inhibition of autophagy and anti-apoptotic mechanisms that are important for the survival of immune memory cells. However, we found that memory T cells and memory B cells are preserved in mice after SECH treatments. The induction of anti-apoptotic molecules and autophagy during latency reversal likely confers the resistance of uninfected cells to agents that target these pro-survival mechanisms (**Figure 5B**). Therefore, SECH does not seem to cause the depletion of immune memory cell population which would be detrimental to the maintenance of immunological memory. Consistent with the *in vivo* studies of T cell subsets in HIV-1-infected humanized mice, we have previously shown that *in vitro* SECH treatments cleared HIV-1 reservoirs in PBMCs from ART-naïve or ART-experienced HIV-1 patients (27). Moreover, T cell subsets, including naïve, central memory and effector memory T cells, were preserved after *in vitro* SECH treatments (27), supporting the specificity for SECH in targeting HIV-1-infected cells, but not uninfected bystander cells.

Recent evidence indicates that latent HIV-1 proviruses can be detected in CD4^+^ T cells at different development stages (35–38). Consistently, we found that HIV-1 latent infection could be established in naïve and memory T cells. Homeostatic proliferations of T cell reservoirs may account for the expansion of certain integrated HIV-1 provirus (20, 46–48). We observed that latent HIV-1 could be detected in naïve and memory T cells in humanized mice after ART treatments. In mice with successful viral clearance, HIV-1 was not detectable in sorted naïve and memory T cells. This study suggests that SECH is effective in targeting HIV-1 reservoirs in different subsets of T cells, while preserving uninfected cells. The SECH approach may be developed as an effective therapy for treating people living with HIV-1.

## Data availability statement

The raw data supporting the conclusions of this article will be made available by the authors, without undue reservation.

## Ethics statement

The animal study was reviewed and approved by Institutional Animal Care and Use Committee of the Houston Methodist Research Institute.

## Author contributions

Study design and conceptualization were performed by ML, MC, and JW. The experimental protocols were carried out by ML, MC and MB. The data analysis was performed by ML and JW. Original draft was prepared by JW and ML. Data interpretation and presentation were performed by all listed authors. All authors contributed to the article and approved the submitted version.
