# Supplementary material for: Targeting HIV-1 reservoirs in T cell subsets
Source: Front Immunol. 2023 Jan 20;14:1087923. doi: 10.3389/fimmu.2023.1087923 (PMC9895780; doi:10.3389/fimmu.2023.1087923)
Supplement: Supplementary file 1 [file DataSheet_1.pdf]

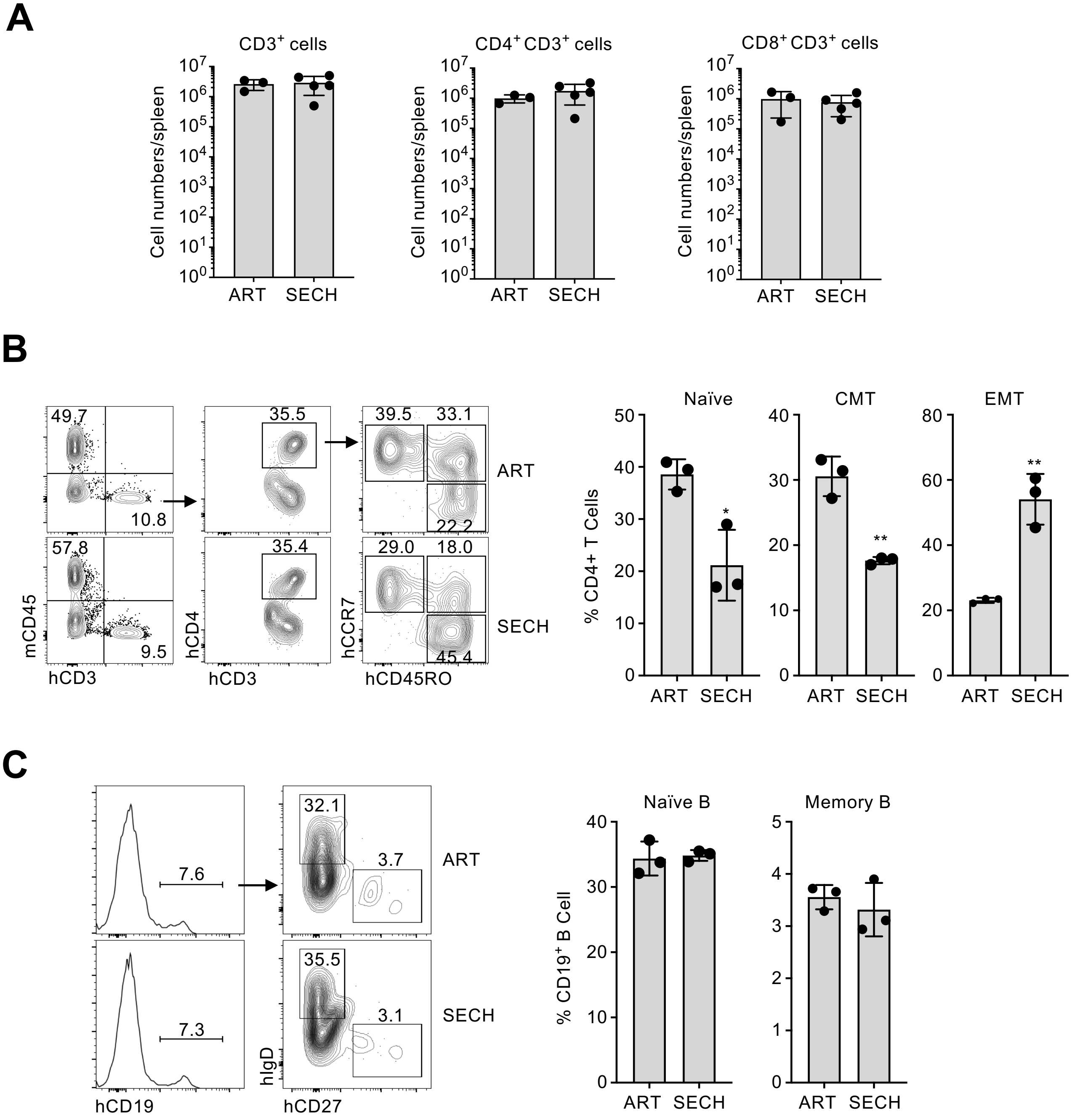

Supplementary Figure 1 | Human T cell subsets in HIV-1-infected Hu-HSC mice after SECH treatments. (**A**) HIV-1-infected Hu-HSC mice were treated by SECH (n=3) or ART alone (n=3). The human immune cells from the spleen were determined and quantified by flow cytometry after withdrawal of treatments. (**B**) Human $CD4^+$ T cell subsets from ART- or SECH-treated mice, including $CCR7^+CCD45RO^-$ naïve cells, $CCR7^+CCD45RO^+$ CMT and $CCR7^-CCD45RO^+$ EMT, were quantified by flow cytometry analyses. (**C**) Human $CD19^+IgD^+CD27^-$ naïve B cells and $CD19^+IgD^-CD27^+$ memory B cells from ART- or SECH-treated mice were quantified after withdrawal of treatments. Data are presented as mean ± SD. * $P< 0.05$ and ** $P< 0.01$.

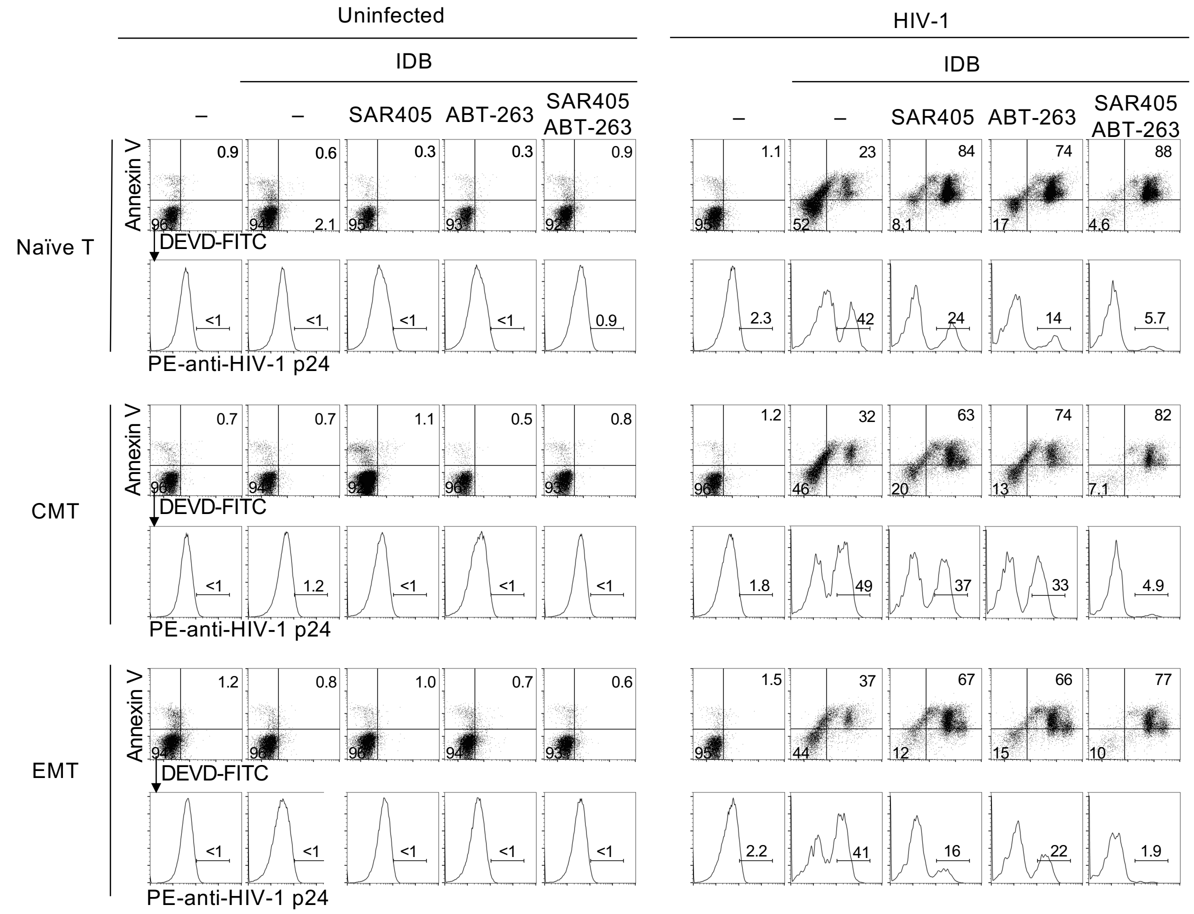

Supplementary Figure 2 | Naïve, central memory (CMT) and effector memory T cells (EMT) were sorted from human PBMCs by flow cytometry. The cells were infected with HIV-1 NL4-3 (1 MOI), followed by culture for 4 days to establish latent infections. Latently infected cells were stimulated with 0.1 μM IDB ABT-263 (0.2 mM) and SAR405 (2 mM) were added as indicated. The cells were cultured for 48 h, followed by incubation with DEVD-FITC, staining with APC-Annexin V and intracellular staining with PE-anti-HIV p24.
